# A Parametric Study of Nonlinear Seismic Response Analysis of Transmission Line Structures

**DOI:** 10.1155/2014/271586

**Published:** 2014-07-15

**Authors:** Li Tian, Yanming Wang, Zhenhua Yi, Hui Qian

**Affiliations:** ^1^School of Civil and Hydraulic Engineering, Shandong University, Jinan 250061, China; ^2^School of Civil Engineering, Zhengzhou University, Zhengzhou 450001, China

## Abstract

A parametric study of nonlinear seismic response analysis of transmission line structures subjected to earthquake loading is studied in this paper. The transmission lines are modeled by cable element which accounts for the nonlinearity of the cable based on a real project. Nonuniform ground motions are generated using a stochastic approach based on random vibration analysis. The effects of multicomponent ground motions, correlations among multicomponent ground motions, wave travel, coherency loss, and local site on the responses of the cables are investigated using nonlinear time history analysis method, respectively. The results show the multicomponent seismic excitations should be considered, but the correlations among multicomponent ground motions could be neglected. The wave passage effect has a significant influence on the responses of the cables. The change of the degree of coherency loss has little influence on the response of the cables, but the responses of the cables are affected significantly by the effect of coherency loss. The responses of the cables change little with the degree of the difference of site condition changing. The effect of multicomponent ground motions, wave passage, coherency loss, and local site should be considered for the seismic design of the transmission line structures.

## 1. Introduction

Transmission line structures are very important to electric engineering. Most of the transmission lines cross the highly seismic region. The past earthquakes indicate that transmission lines are often damaged under earthquake loading. About 100 transmission lines of Los Angeles city were destroyed in the 1992 Landers earthquake [1]. 38 transmission lines were broken during the 1995 Kobe earthquake [2]. Many transmission lines were pulled off in the 2008 Wenchuan earthquake [3]. A lot of transmission lines were ruptured during the 2013 Lushan earthquake. To guarantee the safety of transmission lines during earthquake, the parametric study of nonlinear responses of the transmission line structures under earthquake loading should be accurately obtained.

Most of research has focused on the actions of static load, impulsive load, and equivalent static wind load. There are no calculation methods about how to consider the transmission line structures under earthquake loading in current seismic codes [4, 5]. Some research has been performed to analyze the seismic responses of transmission tower and transmission line under earthquake loading. Li et al. [6, 7] have completed a number of studies on seismic effects on transmission towers and have verified that the effect of transmission lines in seismic design should not be neglected.

With the development of techniques, the spans of transmission lines have increased dramatically. It is unrealistic to assume that earthquake ground motions for long span transmission tower-line system are the same and single component. Ghobarah et al. [8] investigated the effects of multisupport excitations on the response of overhead power transmission tower and line. The results indicated that the assumption of uniform ground motions at all supports of a transmission line does not provide the most critical case for the response calculations. Tian et al. [9] studied the behavior of power transmission tower-line system subjected to spatially varying ground motions. The effects of the incident angle of the seismic wave, coherency loss, and wave travel on the transmission tower are investigated. Li et al. [10, 11] investigated the response of a transmission tower-line system at a canyon site to spatially varying ground motions. The results showed that the effect of ground motion spatial variations should be incorporated in seismic analysis of the transmission tower-line system. In addition, Tian et al. [12] analyzed the effect of multicomponent multisupport excitations on the response of the transmission tower-line system. Multiple effect parameters were considered, but the responses of the transmission tower were obtained only. Wang et al. [13] researched the progressive collapse analysis of a transmission tower-line system under earthquake. The results indicated that the proposed procedure can provide collapse mode and vulnerable points for use in seismic performance and retrofit evaluation of structure. Furthermore, Tian et al. [14] studied seismic responses of straight line type and broken line type transmission tower-line systems subjected to nonuniform seismic excitations. The results showed that the effect of nonuniform ground motions should be considered in seismic design for the straight line type and broken line type transmission lines practical engineering. The previous study concluded that the responses of transmission tower under nonuniform and multicomponent ground motions were different from that of under uniform and single ground motion. A lot of studies about the response of transmission towers are obtained, but there is little research about the parametric study of nonlinear response of transmission line structures under earthquake loading.

The parametric studies of seismic response analysis of transmission line structures considering geometric nonlinearity subjected to earthquake loading are carried out in this paper. The transmission lines are modeled by cable element account for the nonlinearity of the cable based on a real project. The effects of multicomponent ground motions, correlations among multicomponent ground motions, wave travel, coherency loss, and local site on the responses of the cables are investigated using nonlinear time history analysis method, respectively. The analysis results could provide reference for the seismic design of the transmission line structures.

## 2. Transmission Line Structures Model

**Figure 1:**
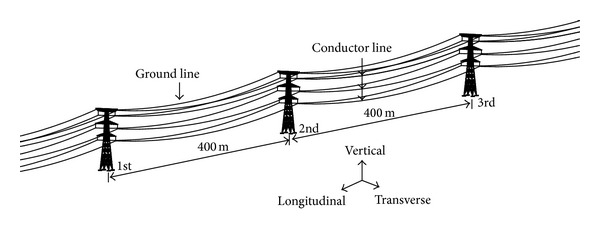
Finite element model of transmission line structures.

A typical three-dimensional finite element model of transmission line structures is established based on a real electric project in the north of China. SAP2000 finite element program is used to simulate the transmission line structures. As shown in Figure 1, the transmission line structures include three towers and four span conductors lines and ground lines. Figure 1 shows the conductor and ground lines. The longitudinal, transverse, and vertical directions of the transmission line structures are shown in Figure 1. Conductor line and ground line properties are shown in Table 1. The transmission line is modeled by 40 two-node isoparametric cable elements with three translational degrees of freedom at each node. The upper one layer line is ground cable, and the lower three layer lines are four-bundled conductor cables. The distance between adjacent transmission towers is 400 m. The connections between transmission towers and transmission lines are hinged using insulators. The side spans of the transmission lines are hinged at the same height of middle transmission tower.

**Figure 2:**
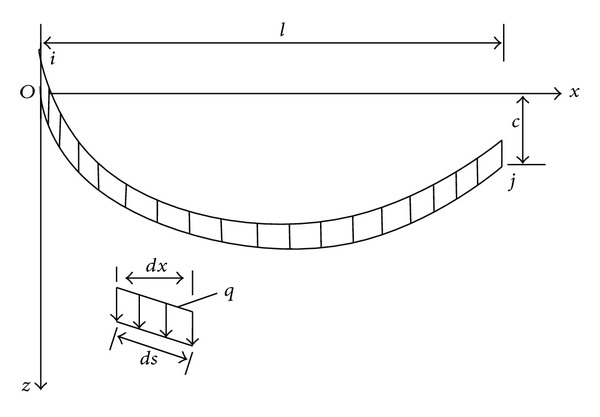
Coordinates of a single cable under self-weight.

Under self-weight, the cables' configuration is a catenary. Based on the coordinate system illustrated in Figure 2, (1) was used to define the initial geometry of the cable profile [15]

(1) $$\begin{array}{c} z=\frac{H}{q}\left|\cosh\left(\alpha \right)-\cosh\left|\frac{2\beta x}{l}-\alpha \right|\right|, \end{array}$$

where *α* = sinh^−1^|*β*(*c*/*l*)/sin(*β*)| + *β*, *β* = *ql*/2*H*, *H* represents the initial horizontal tension which can be obtained from a preliminary static analysis, and *q* denotes the uniformly distributed gravity loads along the conductor and ground lines.

## 3. Simulation of Nonuniform Ground Motions

An empirical coherency loss function derived from SMART-1 array is used in the paper [16]. The coherency loss function between two points *i* and *j* is

(2) $$\begin{array}{c} \left|\gamma_{ij}\left(\omega ,d_{ij}\right)\right|=\exp-\left(\beta d_{ij}\right)\cdot \exp\left\{-a\left(\omega \right)\sqrt{d_{ij}}{\left(\frac{\omega }{2\pi }\right)}^{2}\right\} \end{array}$$

in which *d*_*ij*_ is the projected distance in the wave propagation direction between points *i* and *j* in the wave propagation direction, *β* is a constant, and *a*(*ω*) is a function with the form

(3) a(ω)={2πaω+bω2π+c,0.314 rad/s≤ω≤62.83 rad/s,0.1a+10b+c,ω≥62.83 rad/s,

where the constants *a*, *b*, and *c* can be obtained by least-squares fitting the coherency function of recorded motions. The constants in coherency function are *a* = 3.583 × 10^−3^, *b* = −1.811 × 10^−5^, *c* = 1.177 × 10^−4^, and *β* = 1.019 × 10^−4^, which were obtained by processing recorded motions during Event 45 at the SMART-1 array [16], and it represents highly correlated ground motions. To compare the change of the coherency loss, different degrees of coherency loss are selected based on Bi et al.'s studies [17].

**Figure 3:**
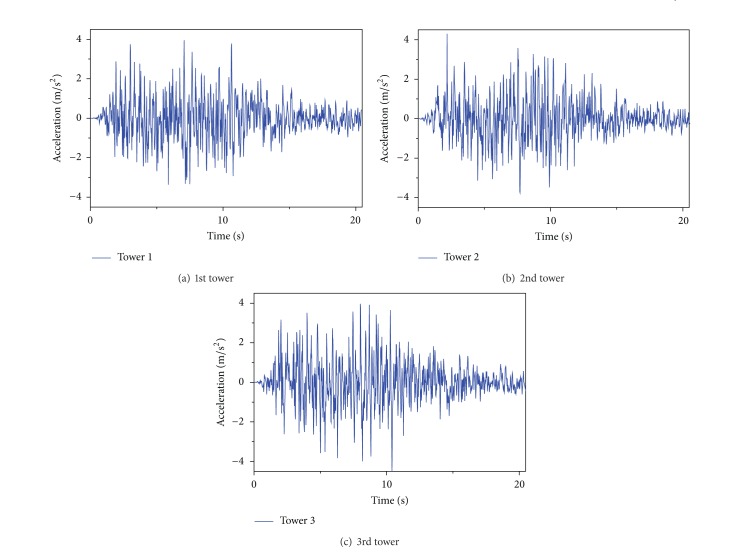
Acceleration time histories of three tower points in longitudinal direction.

A stochastic approach based on random vibration analysis is used, and the simulated ground motion time history is iterated to be compatible with the response spectrum defined in Code for Design of Seismic of Electrical Installations. Reference [18] gives the parameters of Clough-Penzien model according to the Code for Design of Seismic of Electrical Installations. The transmission cable structures are assumed to locate in the mid-firm soil. The peak ground motion of the longitudinal component is 0.4 g. The intensities of the transverse component and vertical component, as stated in the code, are 0.85 and 0.65 times of the longitudinal component, respectively. The three components of the ground motion are assumed to coincide with the principal axes. The three components of ground motions along a set of principal axes are uncorrelated based on Penzien and Watabe's studies [19].  Figure 3 shows acceleration time histories of three transmission tower points in longitudinal direction on mid-form soil with apparent velocity 1000 m/s.

## 4. Numerical Simulation and Discussion

The parametric studies of nonlinear seismic responses of the transmission line structures under earthquake loading are analyzed using nonlinear time history analysis method. The geometric nonlinearity is taken into account due to large deformation of the transmission lines. The HHT (Hilber-Hughes-Taylor) method is applied in the numerical integration. The layers of cables shown in Figure 1 from upper to down are numbered 1, 2, 3, and 4, respectively.

### 4.1. Effect of Multicomponent Ground Motions

To study the effect of multicomponent ground motions, three typical natural seismic waves are selected, which are El Centro wave, Oka wave, and Taft wave. The selection of seismic waves is shown in Table 2. Three components of the natural seismic waves are considered in the paper. The direction of the maximum acceleration component of the horizontal seismic wave is denoted by the horizontal 1, while the other direction component of the horizontal seismic wave is denoted by the horizontal 2, and the vertical component of seismic wave is denoted by vertical. The maximum acceleration value of the ground motion is adjusted to 0.4 g, and the other two directions are scaled according to the proportion.

Four cases are considered, longitudinal excitation only (Case 1), transverse excitation only (Case 2), vertical excitation only (Case 3), and multicomponent excitations (Case 4). Case 1 is longitudinal excitation only, and the horizontal 1 component of the seismic wave is inputted along longitudinal direction of the transmission line structures model. Case 2 is transverse excitation only, and the horizontal 2 component of the seismic wave is inputted along transverse direction of the transmission line structures model. Case 3 is vertical excitation only, and the vertical component of the seismic wave is inputted along vertical direction of the transmission line structures model. Case 4 is multicomponent excitations, and the horizontal 1, horizontal 2, and vertical component of seismic wave are inputted together along longitudinal, transverse, and vertical direction of the transmission line structures model, respectively.

**Figure 4:**
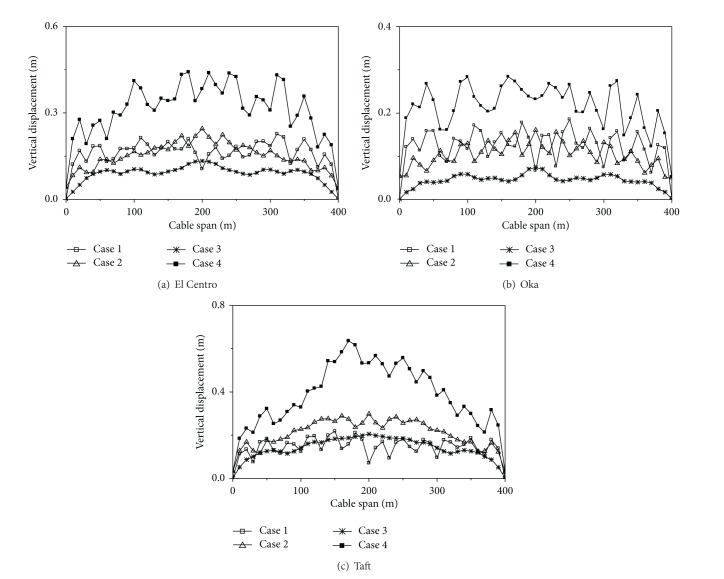
Vertical displacements of the cable under different analysis cases.

The maximum value curves of the vertical displacements of the cable under different analysis cases are shown in Figure 4. It can be seen from Figure 4 that the vertical displacement of the cable under longitudinal or transverse excitation only is larger than that of under vertical seismic excitation only, so the longitudinal or transverse excitation has a great influence on the response of the vertical displacement of the cable. The vertical displacements of the cable under multicomponent excitations are significantly larger than that of under vertical, longitudinal, or transverse excitation only. The longitudinal and transverse seismic excitations have a large coupling with the response of the vertical displacement of the cable. Therefore, multicomponent seismic excitations should be considered for the transmission line structures.

### 4.2. Effect of Correlations among Multicomponent Ground Motions

To research the effect of the correlations among multicomponent ground motions, four cases are considered, uniform (Case 1), *α* = 0° (Case 2), *α* = 18° (Case 3), and *α* = 45° (Case 4). The correlations among multicomponent ground motions are selected based on previous studies [12].

The maximum values of the tension forces of the cables under different degrees of the coherence are shown in Table 3. It can be seen from Table 3 that the tension forces of the cables have an increasing tendency with the increasing of the degree of the coherence. Ignoring the correlations among the multicomponent ground motions, the results may be small, but the changes are very little. The above analysis indicates that the effect of correlations among multicomponent ground motions could be neglected.

### 4.3. Effect of Ground Motion Spatial Variations

To investigate the effect of ground motion spatial variations, four cases are considered, uniform (Case 1), wave passage effect only (Case 2), coherency loss effect only (Case 3), and local site effect only (Case 4). Case 1 is the uniform excitation, because the apparent velocity, coherency loss, and soil condition of ground motion are assumed to be infinite, highly correlated, and mid-firm site, respectively.

**Figure 5:**
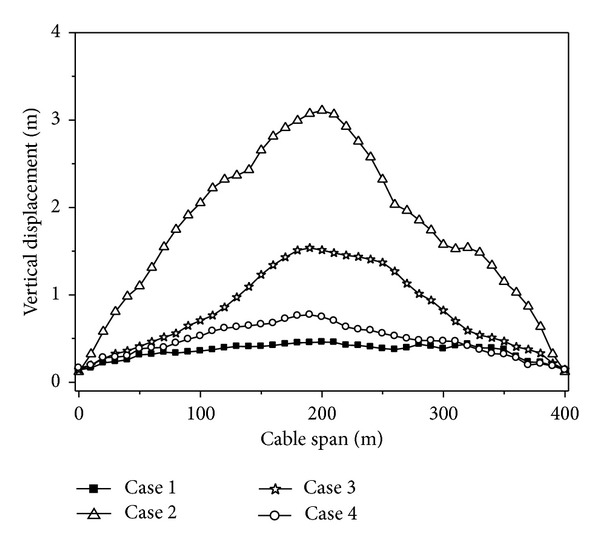
Vertical displacements of the cable under different analysis cases.

The maximum value curves of the vertical displacements of the cable under different analysis cases are shown in Figure 5. It can be seen from Figure 5 that the vertical displacements of the cable considering wave travel effect only, coherency loss effect only, or local site effect only are larger than that of under uniform excitation. The vertical displacements of the cable considering wave travel effect only are larger than that of considering coherency loss effect only or local site effect only. Existing research [20] has shown that the wave travel effect is very important to the responses of structure when the structure is flexible, and the responses are mainly decided by dynamic response of the structure. The coherency loss effect is very important to the responses of structure when the structure is rigid, and the responses are mainly decided by quasistatic response of the structure. Therefore, wave travel effect of ground motion is more obvious to the influence of the structure than the other effect for the flexible structure of the transmission lines.

### 4.4. Wave Travel Effect

To study the effect of apparent velocity, ten different velocities of wave propagation are considered in the analysis, uniform (Case 1), 200 m/s (Case 2), 400 m/s (Case 3), 600 m/s (Case 4), 800 m/s (Case 5), 1000 m/s (Case 6), 1200 m/s (Case 7), 1600 m/s (Case 8), 2000 m/s (Case 9), and 3000 m/s (Case 10), to cover the range of practical propagation velocities in engineering. In all these cases, the coherency loss and soil condition of ground motion are assumed to be highly correlated and the mid-firm site, respectively.

**Figure 6:**
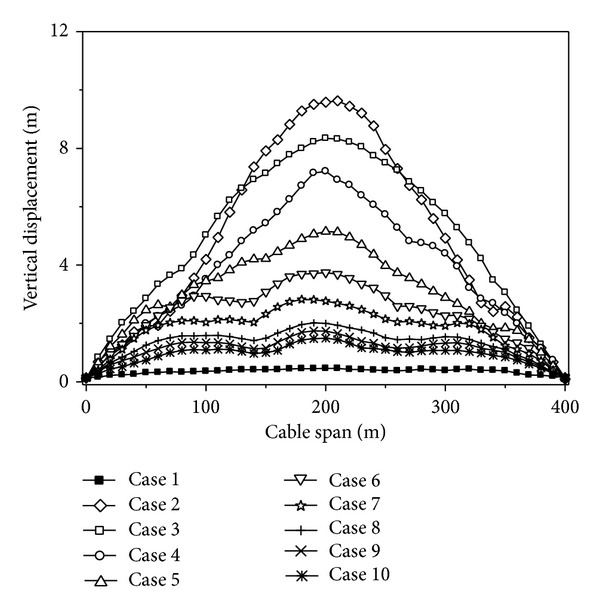
Vertical displacements of the cable under different traveling wave velocities.

The maximum value curves of the vertical displacements of the cable under different traveling wave velocities are shown in Figure 6. It can be seen from Figure 6 that the vertical displacements of the cable increase with the decreasing wave velocity. The maximum vertical displacements of the cable appear when the velocity is 200 m/s. The vertical displacements of the cable decrease with the increasing wave velocity, but it is larger than that of under uniform excitation. Therefore, the vertical displacement of the cable is very sensitive to the traveling wave velocity of the seismic wave.

**Figure 7:**
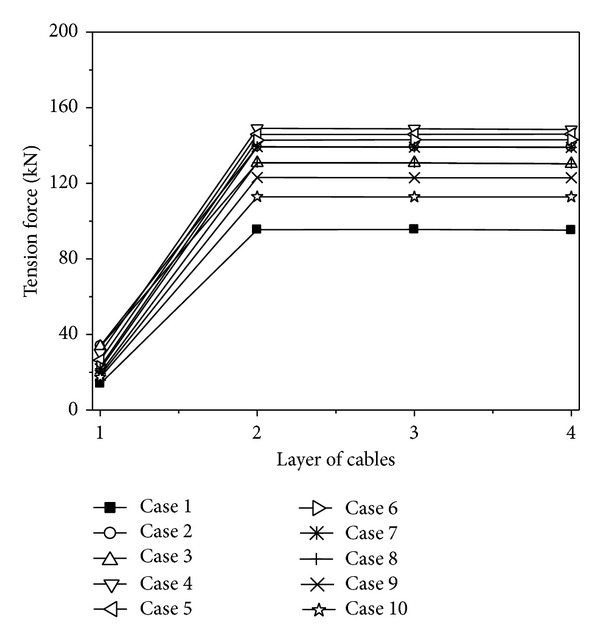
Tension forces of the cables under different traveling wave velocities.

The maximum value curves of tension forces of the cables under different traveling wave velocities are shown in Figure 7. The tension forces of the cables change very little when the traveling wave velocity is less than 200 m/s, but it is larger than that of under uniform excitation. With the traveling wave velocity increasing, the tension forces of the cables decrease gradually. Neglecting the wave passage effect of ground motion, the maximum tension forces of the cables could be underestimated by more than 50%.

Based on the variations of the displacements and tension forces of the cables considering the change of traveling wave velocity, the wave travel effect has a significant influence on the response of the cables. The vertical displacements of the cable are amplified greatly considering the wave travel effect. The vibration of the cable is very large, which would lead to discharge and short circuit. The tension forces of the cables considering the wave travel effect are larger than that of under uniform excitation. Because the tension forces of the cables are too large, the transmission lines would be pulled off and the situations usually occur in the past earthquakes. Therefore, it is necessary to estimate the traveling wave velocity accurately.

### 4.5. Coherency Loss Effect

To investigate the effect of coherency loss, uniform (Case 1), uncorrelated (Case 2), weakly (Case 3), intermediately (Case 4), highly (Case 5), and completely correlated (Case 6) ground motions are considered, respectively. It should be noted that the correlation as low as uncorrelated does not usually occur at short distances, unless there are considerable changes in the local geology from one support to the other. In all these cases, the apparent velocity and soil condition of ground motion are assumed to be 1000 m/s and the mid-firm site, respectively.

**Figure 8:**
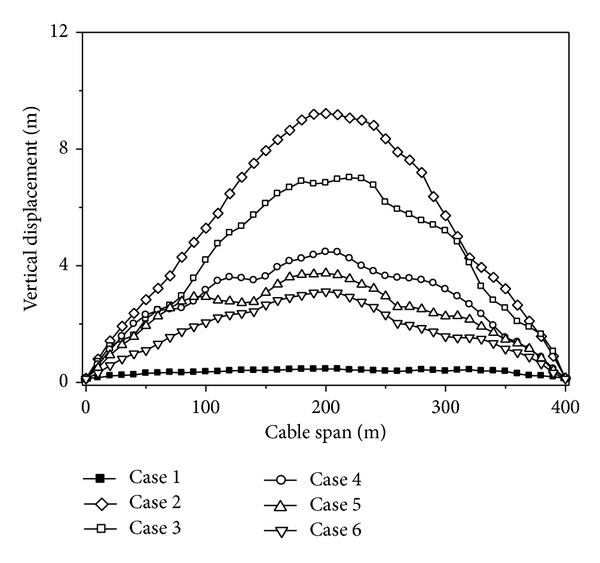
Vertical displacements of the cable under different degrees of coherency loss.

The maximum value curves of the vertical displacements of the cable under different degrees of coherency loss are shown in Figure 8. It can be seen from Figure 8 that the maximum vertical displacements of the cable appear when the coherency loss is uncorrelated. The vertical displacements of the cable have an increasing tendency with the decrease of the degree of coherency loss. The changes of the vertical displacements are very little when the coherency losses are intermediately, highly, and completely correlated.

**Figure 9:**
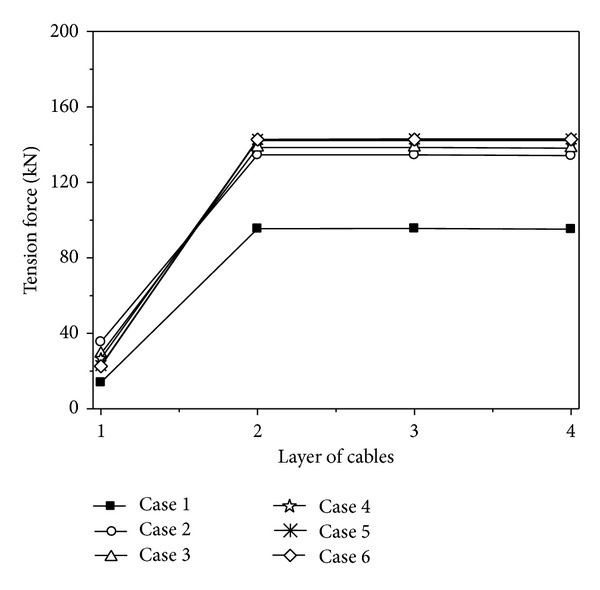
Tension forces of the cables under different degrees of coherency loss.

The maximum value curves of tension forces of the cables under different degrees of coherency loss are shown in Figure 9. It can be seen from Figure 9 that the change of coherency loss has little influence on the tension forces of the cables, so the change of coherency loss can be ignored. The tension forces of the cables considering coherency loss effect are larger than that of under uniform excitation, so the effect of coherency loss should be considered. Neglecting the coherency loss effect of ground motion, the maximum tension forces of the cables could be underestimated by more than 50%.

The variations of the displacement and force responses of the cables considering the change of coherency loss can be obtained from the above analysis. The vertical displacements of the cable have an increasing tendency with the decrease of the degree of coherency loss. The change of coherency loss can be ignored, but the effect of coherency loss must be considered. Therefore, it is very important to consider the coherency loss effect of ground motion for the seismic design of the transmission line structures.

### 4.6. Local Site Effect

To research the effect of local site influence on the cable responses, eight cases are considered, Case 1~Case 8. Analysis cases considering the effect of local site are shown in Table 4. Mid-firm, mid-soft, and soft sites are denoted by F, MF, MS, and S, respectively. In all these cases, the apparent velocity and coherency of ground motion are assumed to be 1000 m/s and highly correlated, respectively.

**Figure 10:**
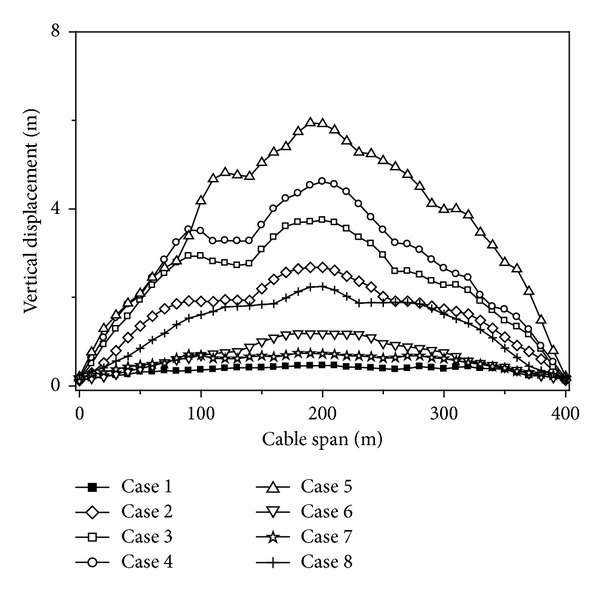
Vertical displacements of the cable under different site conditions.

The maximum value curves of the vertical displacements of the cable under different site conditions are shown in Figure 10. It can be seen from Figure 10 that the vertical displacements of the cable have an increasing tendency with the site condition growing soft. The vertical displacements of the cable increase with the degree of the difference of site condition increasing.

**Figure 11:**
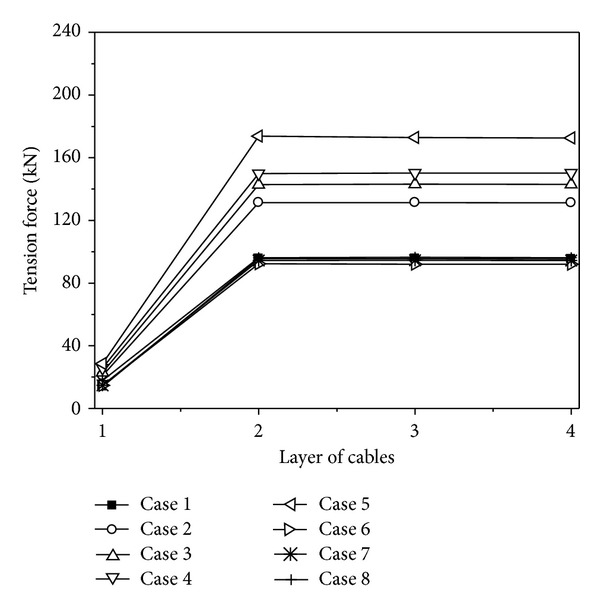
Tension forces of the cables under different site conditions.

The maximum value curves of tension forces of the cables under different site conditions are shown in Figure 11. It can be seen from Figure 11 that the tension forces of the cables have an increasing tendency with the site condition growing soft, and the maximum tension forces of the cables appear when the three transmission towers are located on soft sites. The tension forces of the cables change very little when the site is located in different types, and it could be ignored.

Based on the above analysis, the variations of the displacement and tension force responses of the cables considering different site conditions can be summarized. The vertical displacements and tension forces of the cable have an increasing tendency with the site condition growing soft. The responses of the cables change little with the degree of the difference of site condition changing, especially for the tension forces of the cables. Therefore, the local site effect should be considered for the seismic design of the transmission line structures.

## 5. Conclusion

The parametric studies of nonlinear dynamic responses of the transmission line structures subjected to earthquake loading are investigated in the paper. The effects of multicomponent ground motions, correlations among multicomponent ground motions, ground motion spatial variation, wave passage, coherency loss, and local site on the transmission line structures are considered, respectively. Based on the numerical results, the following conclusions are drawn.

1. The vertical displacements of the cable under multicomponent excitations are significantly larger than that of under vertical, longitudinal, or transverse excitation only. Multicomponent seismic excitations should be considered.
2. Ignoring the correlations among the multicomponent ground motions, the response of the cable may be small, but the changes are very little. The correlations among multicomponent ground motions can be neglected.
3. The responses of the cables considering the effect of ground motion spatial variations are larger than that of under uniform excitation. Wave travel effect of ground motion is more obvious to the influence of the structure than the other effect for the flexible structure of the transmission line structures.
4. The wave passage effect has a significant influence on the responses of the cables. Neglecting the wave passage effect in analysis, the cables responses would be underestimated. Because the tension forces of the cables are too large, the transmission lines would be pulled off. It is necessary to estimate the traveling wave velocity accurately.
5. The change of the degree of coherency loss has little influence on the response of the cables. The responses of the cables are affected significantly by the effect of coherency loss. It is very important to consider the coherency loss effect of ground motion for the seismic design of the transmission line structures.
6. The vertical displacements and tension forces of the cables have an increasing tendency with the site condition growing soft. The responses of the cables change little with the degree of the difference of site condition changing, especially for the tension forces.

## Figures and Tables

**Table 1:** Conductor line and ground line properties.

Type	Conductor line	Ground line
Transmission line	LGJ-400/35	LGJ-95/55
Outside diameter (m)	26.82*E* − 3	16.00*E* − 3
Modulus (GPa)	65	105
Transversal cross-section (m^2^)	425.24*E* − 6	152.81*E* − 6
Mass per unit length (Kg/m)	1.3490	0.6967
Expansion coefficient (1/°C)	2.05*E* − 005	1.55*E* − 005

**Table 2:** Selection of seismic wave.

Number	Earthquake	Event date	Magnitude	Station
①	Imperial Valley	May 18, 1940	6.7	El Centro
②	Kobe	January 16, 1995	6.9	Oka
③	Kern County	July 21, 1952	7.4	Taft

**Table 3:** Tension forces of the cables under different degrees of the coherence (kN).

Layer	Case 1	Case 2	Case 3	Case 4
①	13.98	22.97	23.21	23.91
②	95.51	142.80	143.12	143.79
③	95.52	143.13	143.77	144.22
④	95.20	143.01	143.63	143.91

**Table 4:** Analysis cases considering the effect of local site.

Case	Apparent velocity	Coherency	Soil condition		
			1st tower	2nd tower	3rd tower
Case 1	Infinite	Perfectly	MF	MF	MF
Case 2	1000 m/s	Highly	F	F	F
Case 3	1000 m/s	Highly	MF	MF	MF
Case 4	1000 m/s	Highly	MS	MS	MS
Case 5	1000 m/s	Highly	S	S	S
Case 6	1000 m/s	Highly	F	MF	F
Case 7	1000 m/s	Highly	MS	MF	MS
Case 8	1000 m/s	Highly	S	MF	S
